# The heart of the matter: in search of causal effects of depression on somatic diseases

**DOI:** 10.1186/s12916-018-1144-1

**Published:** 2018-08-23

**Authors:** Annelieke M. Roest, Peter de Jonge

**Affiliations:** Department Developmental Psychology, Faculty Behavioral and Social Sciences, Interdisciplinary Center Psychopathology and Emotion Regulation, University of Groningen, Groningen, The Netherlands

## Abstract

The question of whether depression is a causal factor in somatic diseases remains unanswered despite decades of research. In an extensive umbrella review of systematic reviews and meta-analyses, Machado et al. (*BMC Medicine* 16:112, 2018) found significant associations between depression and all-cause and cause-specific mortality. However, as the authors clearly argued, these results do not prove causation. The next logical step is to study the potential effect of depression and other mental disorders by placing emphasis on temporality (associations over time) and specificity (unique associations) of associations between a comprehensive set of mental disorders and somatic diseases. A data-driven approach in large samples could uncover disease development trajectories to provide a route for researchers and clinicians to improve medical outcomes in vulnerable patient groups.

## Background

Depression is a major health problem, being among the top five causes of burden of disease in Europe and North America according to the Global Burden of Disease study [1]. This hierarchical list of diseases is based on disability-adjusted life years, namely years of life lost due to premature mortality and years lived with disability, attributed to all diseases. Since only direct causes of death are taken into account, with suicides coded under injuries, the high ranking of depression is solely due to the high number of years lived with disability. Yet, if depression also affects mortality by causing other diseases, the Global Burden of Disease study estimates for depression, and mental disorders in general, significantly underestimate the burden attributable to these disorders.

### Depression as a potential cause of all-cause and cause-specific mortality

Many studies have described the putative association between depression and mortality; while the association is most obvious in the case of completed suicide, a substantial amount of research has shown the possibility that depression increases the risk of somatic diseases, thereby indirectly leading to death. In their work in *BMC Medicine*, Machado et al. [2] provide a comprehensive umbrella review on systematic reviews and meta-analyses examining the association between depression and all-cause and cause-specific mortality, confirming the positive associations that have been previously reported for all included causes of mortality. However, the authors warn against concluding that these associations are causal, given that the strength of the evidence was reduced to ‘suggestive’ when considering only studies that examined depressive disorder (instead of depressive symptoms) or that accounted for potential confounders. Their paper shows that the question of whether the association between depression and mortality is causal is an important topic, yet, after decades of research, it remains unanswered at best.

### Intervention trials

Ultimate proof for a causal association between depression and somatic diseases could be provided by randomized controlled trials in which medical outcomes, e.g., survival, are positively influenced by treating depression. This type of trial has been conducted in specific patient samples such as individuals with cardiac disease; nevertheless, these trials led to disappointing results as no effects of antidepressant treatment on cardiac outcomes have been observed (e.g. [3, 4]). However, in secondary analyses, such as in subgroups of participants for example, positive results have been obtained [5]. In addition, several studies have suggested potential explanations for the negative findings, including treatment resistance of specific (somatic/affective) depressive symptoms [6, 7]. Although it could be argued that the development of interventions for these persistent depressive symptoms could improve the medical prognosis of patients with cardiac disease [7], confounding by somatic disease processes may also be a reasonable explanation of these results.

### Specific and temporal associations

Other approaches that can be taken to examine the likelihood of causality is to assess the specificity of associations and temporal associations between diseases [8]. Machado et al. [2] took an important step regarding the specificity of the effect of depression by examining all-cause and cause-specific mortality in different populations such as in patients with cancer or heart failure. Specific associations between depression and outcomes could suggest causal effects, yet the authors did not find particularly strong associations for any of the specific diseases [2]. However, it can be questioned whether mechanisms linking depression to somatic diseases are in fact specific since suggested pathways include general mechanisms, such as inflammatory processes and health behaviors, linked to various somatic diseases. In that respect, it is also important to note that studies on the association between depression and somatic diseases in general try to control for health behaviors in the best possible way (e.g., [9]). However, health behavior factors, such as smoking, alcohol use, body mass index, and physical inactivity, could also be potential mediators of the association, thereby underestimating the strength of the effects.

Another important factor is the temporality, or order, of the associations. Depression predicts the development of somatic diseases, but these somatic diseases in turn also predict depression. Associations between depression and potential mediating mechanisms, e.g., obesity [10] and physical activity [11], are also bidirectional in nature. Further, although mechanisms are usually studied in isolation, they are in fact closely related, e.g., inflammatory markers and body mass index. Taking a temporal approach in examining the association between depression, other mental disorders, potential mediating mechanisms, and somatic diseases can provide greater insight of potential causal associations between these factors. For example, anxiety disorders precede depression in the majority of cases and could therefore, in principle, explain part of the effect of depression on somatic diseases. Additionally, factors occurring even earlier in life, including a genetic predisposition for both depression and somatic diseases (e.g., by a shared genetic substrate for depression and autonomic dysfunction [12]), could be the driving factor.

### Steps to take

Research on temporality and specificity should now progress by taking multiple somatic diseases and mental disorders into account instead of limiting analyses to one outcome such as cancer or heart disease. A data-driven approach could be used to examine temporal and specific associations between depression, other mental disorders, somatic diseases, and mortality. For example, a study that applied this approach by examining temporal associations between disease diagnoses (mainly somatic) retrieved from electronic health registries covering large numbers of individuals found that gout plays a central role in the progression of cardiovascular diseases and highlighted the important role of retinal disorders as a marker of disease progression in individuals with diabetes [13]. Disease trajectories may also differ between individuals, information that could be used to identify those at risk for a specific outcome. For example, a study on risk of sepsis mortality found that different trajectories, with three major starting points, namely cardiovascular disease, diabetes, and alcohol abuse, led to such an outcome [14].

## Conclusion

It is time to move forward from studying the association between one mental disorder and one somatic disease. These studies are unlikely to provide definitive answers since reality is much more complex. Instead, the temporal order of multiple mental disorders and somatic diseases, ideally including the mediating mechanisms, should be examined in concert. Findings from these studies could demonstrate linkages between specific diseases for which previous results have been inconclusive. Furthermore, trajectories that may be suggestive of specific disease development patterns could be uncovered. However, these studies in themselves are not capable of proving causality per se, but may provide persuasive results on the actual burden of disease resulting from depression and other mental disorders as well as important suggestions on when and how to intervene to reduce this burden.
